# Secondhand Smoking and Obesity Among Nonsmoking Adolescents Aged 12–15 Years From 38 Low- and Middle-Income Countries

**DOI:** 10.1093/ntr/ntaa053

**Published:** 2020-03-25

**Authors:** Ai Koyanagi, Lee Smith, Hans Oh, Lin Yang, Sarah E Jackson, Josep Maria Haro, Jae I I Shin, Andre F Carvalho, Louis Jacob

**Affiliations:** 1 Parc Sanitari Sant Joan de Déu, CIBERSAM, Dr Antoni Pujadas, Barcelona, Spain; 2 ICREA, Barcelona, Spain; 3 The Cambridge Centre for Sport and Exercise Sciences, Anglia Ruskin University, Cambridge, UK; 4 University of Southern California, Suzanne Dworak Peck School of Social Work, Los Angeles, CA; 5 Department of Cancer Epidemiology and Prevention Research, Alberta Health Services, Calgary, Alberta, Canada; 11 Departments of Oncology and Community Health Sciences, Cumming School of Medicine, University of Calgary, Calgary, Alberta, Canada; 6 Department of Behavioural Science and Health, University College London, London, UK; 7 Department of Pediatrics, Yonsei University College of Medicine, Seoul, Republic of Korea; 8 Centre for Addiction & Mental Health (CAMH), Toronto, Ontario, Canada; 9 Department of Psychiatry, University of Toronto, Toronto, Ontario, Canada; 10 Faculty of Medicine, University of Versailles Saint-Quentin-en-Yvelines, Montigny-le-Bretonneux, France

## Abstract

**Introduction:**

Secondhand smoking (SHS) may be a risk factor for obesity in adolescence, but data on the association between SHS and obesity are scarce, especially from low- and middle-income countries (LMICs). Therefore, the aim of this study was to assess the association between SHS and obesity among adolescents aged 12–15 years from 38 LMICs.

**Methods:**

Cross-sectional data from 38 LMICs that participated in the Global School-based Student Health Survey (GSHS) were analyzed. Body mass index was calculated based on measured weight and height. The 2007 WHO Child Growth reference was used to define obesity. SHS was categorized as no exposure, non-daily exposure (ie, 1–6 days), and daily exposure (ie, 7 days) based on the number of days exposed to secondhand smoke in the past 7 days. Multivariable logistic regression and meta-analyses were conducted to assess the associations.

**Results:**

The analyzed sample consisted of 88 209 adolescents aged 12–15 years who never smoked. The overall prevalence of non-daily and daily SHS was 34.2% and 15.7%, respectively. After adjustment for potential confounders, compared with no SHS, there was no significant association between non-daily SHS and obesity (odds ratio [OR] = 0.94; 95% confidence interval [CI] = 0.86–1.02), but adolescents who reported daily SHS were significantly more likely to have obesity (OR = 1.19; 95% CI = 1.06–1.34).

**Conclusions:**

The prevalence of SHS was high among adolescents in LMICs, and daily SHS was associated with a significant increase in odds of obesity. Future studies with longitudinal designs are warranted to assess causality and whether prevention of SHS can reduce the risk of obesity in adolescence.

**Implications:**

In the present large multi-country study on adolescents aged 12–15 years from LMICs, nearly half of the students were exposed to non-daily or daily secondhand smoke. Overall, while non-daily SHS was not significantly associated with obesity, adolescents who reported daily SHS had a significant 1.19 (95% CI = 1.06–1.34) times higher odds of obesity than those who reported no exposure to secondhand smoke. To the best of our knowledge, this is the first multi-country study on SHS and obesity from LMICs, and also the largest study on this topic to date.

## Introduction

Childhood obesity is currently one of the most urgent public health challenges worldwide.^1^ Obesity in childhood often persists into adulthood, and children with obesity are at high risk for cardiovascular diseases at a younger age.^1^ Although factors such as poor diet and lack of physical activity have been identified as drivers of the upward trend in childhood obesity that has occurred over recent decades, other environmental factors may also be important.^2^ In particular, there has been burgeoning evidence that secondhand smoking (SHS) is associated with greater body mass index (BMI) or overweight/obesity in children.^3–9^ For example, the only study which used objective measures of SHS found that children or adolescents with high 4-(methylnitrosamino)-1-(3-pyridyl)-1-butanol (NNAL) levels were nearly twice as likely to have obesity than children with low NNAL levels in the United States.^9^ It has been hypothesized that SHS may lead to greater BMI via inflammation, oxidative stress, and endocrine disruption.^10–12^ Specifically, numerous compounds found in smoke (eg, nicotine, polycyclic aromatic hydrocarbons) have negative endocrine effects that could lead to insulin resistance and metabolic imbalance.^10^ Cigarette smoke may also produce some biological mediators of inflammation through its effect on immune-inflammatory cells,^13^ and in turn, inflammation may increase risk for obesity.^14^ Finally, exposure to cigarette smoke can induce oxidative stress, and this can interact with inflammation to increase risk for obesity.^15^

However, almost all existing studies on SHS and bodyweight have been conducted in high-income countries (HICs), and many of the studies focused only on parental smoking rather than SHS from all sources. This is an important research gap because although the prevalence of obesity is generally higher in HICs,^16^ the vast majority of children with obesity live in low- and middle-income countries (LMICs), as the number of children in LMICs is much greater than in HICs. Furthermore, childhood obesity is increasing more rapidly in LMICs than in HICs. Indeed, the relative increase in obesity among children between 1990 and 2010 has been greater in developing countries (+65%) than in developed countries (+48%).^17^ In addition, tobacco control policy legislation is less prevalent in LMICs compared to HICs, and this may lead to a greater chance of exposure to secondhand smoke in LMICs.^18^ Furthermore, exposure to secondhand smoke can occur in a variety of settings and not only in the household. For example, one study found that among children who reported no smokers in the household, approximately 40% had shown evidence of SHS based on biomarkers.^9^ Intensity of SHS may also differ between HICs and LMICs for differences in factors such as prevalence of smoking, housing conditions pertaining to natural ventilation, crowding at home, and level of enforcement of smoke-free legislation at work and public places.^18^

Thus, the aim of the current study was to assess the association between SHS and obesity among adolescents aged 12–15 years from 38 LMICs spanning six WHO regions using data of the Global School-based Student Health Survey (GSHS).

## Methods

### The Survey

Publicly available data from the GSHS were analyzed. Details on this survey can be found at www.who.int/chp/gshs and www.cdc.gov/gshs. Briefly, the GSHS was jointly developed by the WHO and the US Centers for Disease Control and Prevention (CDC), and other UN allies. The core aim of this survey was to assess and quantify risk and protective factors of major noncommunicable diseases. The survey draws content from the CDC Youth Risk Behavior Survey (YRBS) for which test-retest reliability has been established.^19^ The survey used a standardized two-stage probability sampling design for the selection process within each participating country. For the first stage, schools were selected with probability proportional to size sampling. The second stage involved the random selection of classrooms that included students aged 13–15 years within each selected school. All students in the selected classrooms were eligible to participate in the survey regardless of age. Data collection was performed during one regular class period. The questionnaire was translated into the local language in each country and consisted of multiple-choice response options; students recorded their responses on computer scannable sheets. All GSHS surveys were approved, in each country, by both a national government administration (most often the Ministry of Health or Education) and an institutional review board or ethics committee. Student privacy was protected through anonymous and voluntary participation, and informed consent was obtained as appropriate from the students, parents and/or school officials. Data were weighted for nonresponse and probability selection.

From all publicly available data, we selected all nationally representative datasets from LMICs that included the variables pertaining to this analysis. We excluded countries for which more than 20% of the data on BMI were missing. If there were more than two datasets from the same country, we chose the most recent dataset. Laos was also omitted as estimates for this country could not be obtained due to the low prevalence of SHS and obesity. Thus, a total of 38 countries were included in the current study. The characteristics of each country or survey are provided in Table 1. For the included countries, the survey was conducted between 2003 and 2016, and consisted of 6 low-income, 21 lower middle-income, and 11 upper middle-income countries based on the World Bank classification at the time of the survey for the respective countries. These countries were from six WHO regions: African Region (*n* = 6); Region of the Americas (*n* = 6); Eastern Mediterranean Region (*n* = 10); European Region (*n* = 1); South-East Asia Region (*n* = 7); and Western Pacific Region (*n* = 8).

**Table 1.** Survey Characteristics by Country

Country income	Country	Region	Year	Response rate (%)	*N* (Total)	*N* (nonsmokers)
Low	Afghanistan	EMR	2014	79	1493	1200
	Benin	AFR	2016	78	717	624
	Cambodia	WPR	2013	85	1812	1557
	Myanmar	SEAR	2007	95	2227	2092
	Nepal	SEAR	2015	69	4616	4051
	Uganda	AFR	2003	69	1904	1526
Lower middle	Bangladesh	SEAR	2014	91	2753	2226
	Bolivia	AMR	2012	88	2804	1873
	Djibouti	EMR	2007	83	962	840
	East Timor	SEAR	2015	79	1631	898
	Egypt	EMR	2011	85	2364	2060
	Ghana	AFR	2012	82	1110	721
	Guyana	AMR	2010	76	1973	1258
	Honduras	AMR	2012	79	1486	1080
	India	SEAR	2007	83	7330	6808
	Indonesia	SEAR	2015	94	8806	6767
	Kiribati	WPR	2011	85	1340	762
	Macedonia	EUR	2007	93	1550	1176
	Mongolia	WPR	2013	88	3707	3034
	Morocco	EMR	2010	92	2405	1968
	Pakistan	EMR	2009	76	4998	3980
	Philippines	WPR	2015	79	6162	4590
	Sudan	EMR	2012	77	1401	1132
	Syria	EMR	2010	97	2929	2489
	Tonga	WPR	2010	80	1946	1056
	Vietnam	WPR	2013	96	1743	1582
	Yemen	EMR	2014	75	1553	1091
Upper middle	Algeria	AFR	2011	98	3484	2953
	Costa Rica	AMR	2009	72	2265	1547
	Fiji	WPR	2016	79	1537	1136
	Iraq	EMR	2012	88	1533	1180
	Libya	EMR	2007	98	1891	1637
	Malaysia	WPR	2012	89	16 273	12 944
	Mauritius	AFR	2011	82	2074	1544
	Namibia	AFR	2013	89	1936	1377
	Peru	AMR	2010	85	2359	1539
	Suriname	AMR	2009	89	1046	738
	Thailand	SEAR	2015	89	4132	3173

AFR, African Region; AMR, Region of the Americas; EMR, Eastern Mediterranean Region; EUR, European Region; SEAR, South-East Asia Region; WPR, Western Pacific Region. *N* is based on those aged 12–15 years.

### Obesity (Outcome)

Trained survey staff conducted measurement of weight and height. BMI was calculated as weight in kilograms divided by height in meters squared. Obesity was defined as >2 SDs above the median for age and sex based on the 2007 WHO Child Growth reference.^20^

### SHS (Exposure)

Exposure to secondhand smoke was ascertained by asking, “During the past 7 days, on how many days have people smoked in your presence?” with answer options: 0 day, 1 or 2 days, 3 to 4 days, 5 to 6 days, and all 7 days. Adolescents who replied “0 day” were considered to have no secondhand smoke exposure, while those who were exposed to secondhand smoke in the past 7 days were grouped into the following categories: non-daily (1 to 6 days) and daily (all 7 days). We categorized the SHS variable as such as preliminary analysis showed that daily SHS is particularly strongly associated with obesity.

### Control Variables

These included age, sex, food insecurity (as a proxy for socioeconomic status), physical activity, and fruit/vegetable consumption. As in a previous GSHS study, food insecurity was used as a proxy for socioeconomic status as there were no variables on socioeconomic status in the GSHS.^21^ Specifically, this was assessed by the question, “During the past 30 days, how often did you go hungry because there was not enough food in your home?” Answer options were categorized as “never,” “rarely/sometimes,” and “most of the time/always.” ^22^ To assess levels of physical activity, questions that represented the PACE+ Adolescent Physical Activity Measure^23^ were asked. This measure has been tested for validity and reliability.^23^ The questions asked about the number of days with physical activity of at least 60 minutes during the past 7 days. Those who engaged in ≥5 days of at least 60 minutes of physical activity in a week were considered to have a sufficient amount of physical activity.^24^ Low fruit and vegetable intake was defined as intake of fruit and vegetables less than five times per day (<400 g of fruits and vegetables/day) during the past 30 days.^25^

### Statistical Analysis

The analysis was restricted to adolescents aged 12–15 years as information on the exact age outside of this age range was not available, and the majority of the students were within this age range. Data on 112 252 adolescents aged 12–15 years were available, but the final sample consisted of 88 209 adolescents who had never smoked a cigarette to avoid the confounding effect of tobacco use. Country-wise multivariable logistic regression models, adjusting for age, sex, socioeconomic status (food insecurity), physical activity, and fruit/vegetable consumption were constructed to assess the association between SHS (exposure) and obesity (outcome). The estimates for non-daily SHS (vs. no SHS) and daily SHS (vs. no SHS) were obtained for each country. These estimates were combined into a fixed-effect meta-analysis to obtain an overall estimate (overall and by country-income level). We used fixed-effects rather than random effects to obtain the overall estimate as the level of between-country heterogeneity was low. To assess the level of between-country heterogeneity, the Higgins’s *I*^2^ statistic was calculated. The Higgins’s *I*^2^ represents the degree of heterogeneity between countries that is not explained by sampling error with a value of <40% often considered as negligible and 40%–60% as moderate heterogeneity.^26^ Given that Malaysia had by far the largest sample size, we conducted a sensitivity analysis without Malaysia, to assess whether the results were mainly driven by the inclusion of this country in the analysis. Finally, we also conducted a multivariable linear regression analysis with the continuous BMI variable as the outcome and the number of days exposed to secondhand smoke in the previous 7 days as the exposure variable in its original continuous scale.

All variables were included in the regression analysis as categorical variables with the exception of age (continuous variable). Under 1.4% of the data were missing for the variables used in this study, with the exception of obesity (7.3%). Complete case analysis was done. The sample weighting and the complex study design were taken into account in all analyses with Taylor linearization methods. Results from the logistic regression models are presented as odds ratios (ORs) with 95% confidence intervals (CIs). The level of statistical significance was set at *p* < .05. The statistical analysis was done with Stata 14.1 (Stata Corp LP, College Station, Texas).

## Results

**Figure 1.**
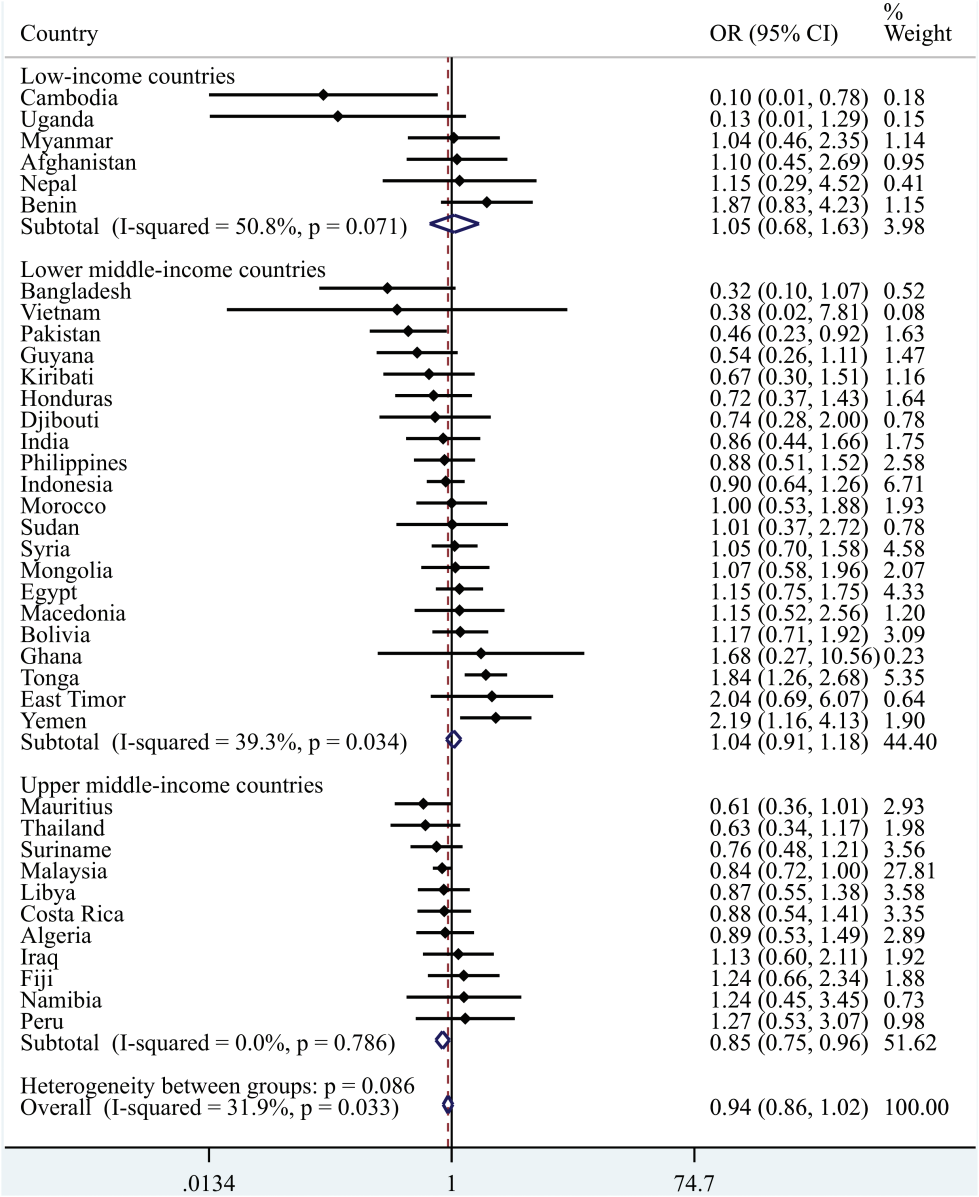
Country-wise association between non-daily secondhand smoking (vs. no secondhand smoking) and obesity estimated by multivariable logistic regression. OR, Odds ratio; CI, Confidence interval. Models are adjusted for age, sex, socioeconomic status (food insecurity), physical activity, and low fruit/vegetable intake. Overall estimate was obtained by meta-analysis with fixed effects.

**Figure 2.**
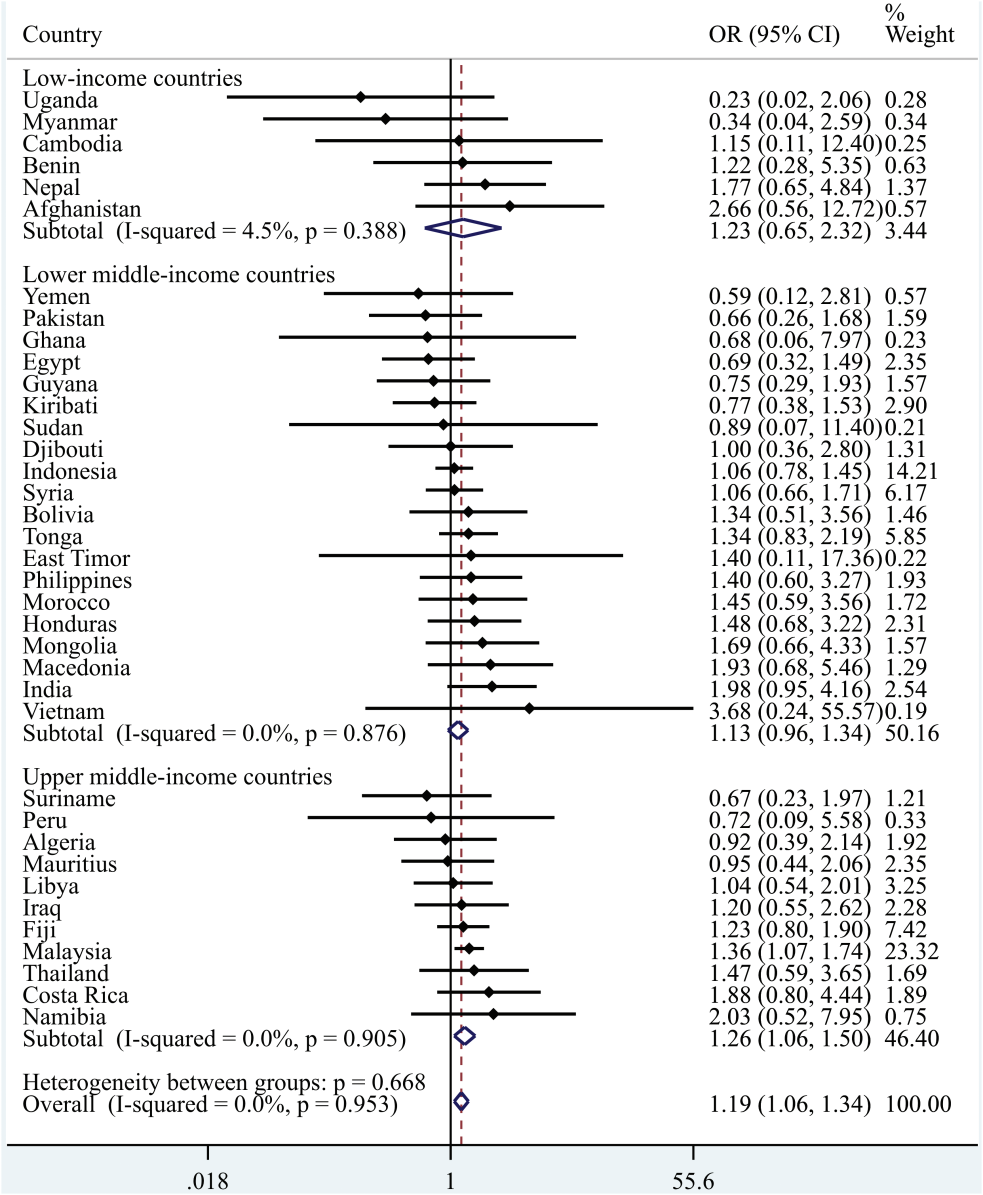
Country-wise association between daily secondhand smoking (vs. no secondhand smoking) and obesity estimated by multivariable logistic regression. OR, Odds ratio; CI, Confidence interval. Models are adjusted for age, sex, socioeconomic status (food insecurity), physical activity, and low fruit/vegetable intake. Overall estimate was obtained by meta-analysis with fixed effects.

The mean (SD) age of the final sample, which only consisted of adolescents who had never smoked a cigarette (*n* = 88 209), was 13.8 (1.0) years and 54.0% were girls. The overall prevalence of obesity was 3.9%, while that of non-daily and daily SHS was 34.2% and 15.7%, respectively, although these figures varied substantially between countries (Table 2). Specifically, the prevalence of obesity ranged from 0.3% in Vietnam to 23.4% in Tonga, while that of daily SHS ranged from 2.6% in Cambodia to 35.4% in Indonesia. The country-wise association between non-daily SHS (vs. no SHS) is shown in Figure 1. Overall, non-daily SHS was not significantly associated with obesity, with the pooled estimate based on a meta-analysis being OR = 0.94 (95% CI = 0.86–1.02) (*I*^2^ = 32%; 95% CI = 0–54). In terms of daily SHS, overall, when compared with no SHS, daily SHS was significantly associated with a 1.19 (95% CI = 1.06–1.34) times higher odds of obesity with no evidence of between-country heterogeneity (*I*^2^ = 0.0%; 95% CI = 0–37) (Figure 2). Estimates obtained by country-income levels were similar. The sensitivity analysis showed that the results for non-daily SHS (vs. no SHS) and daily SHS (vs. no SHS) were similar with or without Malaysia, confirming the fact that the results were not mainly driven by this country, which had a particularly large sample size (data not shown). Finally, the results of the multivariable linear regression analysis showed that a one-day increase in SHS in the past 7 days is associated with a small but significant increase in BMI (Supplementary Figure S1).

**Table 2.** Prevalence of Obesity and Secondhand Smoking Among Nonsmokers By Country

Country income	Country	Region	Obesity	Secondhand smoking	
				Non-daily	Daily
Low	Afghanistan	EMR	2.4	41.8	3.5
	Benin	AFR	2.8	29.1	15.7
	Cambodia	WPR	0.4	41.8	2.6
	Myanmar	SEAR	0.8	43.6	23.1
	Nepal	SEAR	0.5	33.7	17.4
	Uganda	AFR	0.9	24.6	14.9
Lower middle	Bangladesh	SEAR	1.4	19.8	6.5
	Bolivia	AMR	4.3	36.4	3.6
	Djibouti	EMR	4.7	32.7	15.9
	East Timor	SEAR	1.3	49.6	26.0
	Egypt	EMR	7.8	25.6	10.5
	Ghana	AFR	2.1	39.9	12.7
	Guyana	AMR	4.6	34.8	18.1
	Honduras	AMR	6.2	36.4	9.3
	India	SEAR	2.3	29.3	5.9
	Indonesia	SEAR	5.5	38.4	35.4
	Kiribati	WPR	8.3	47.3	24.8
	Macedonia	EUR	3.3	40.7	21.5
	Mongolia	WPR	1.8	45.2	11.3
	Morocco	EMR	2.6	28.4	7.9
	Pakistan	EMR	1.0	32.0	13.5
	Philippines	WPR	3.1	36.4	6.6
	Sudan	EMR	3.2	28.8	6.4
	Syria	EMR	5.7	35.7	23.8
	Tonga	WPR	23.4	36.1	18.1
	Vietnam	WPR	0.3	56.3	17.0
	Yemen	EMR	2.5	33.5	16.4
Upper middle	Algeria	AFR	3.5	29.4	10.4
	Costa Rica	AMR	9.3	35.4	5.9
	Fiji	WPR	8.4	31.0	12.3
	Iraq	EMR	8.6	31.9	13.1
	Libya	EMR	8.1	28.7	13.7
	Malaysia	WPR	9.9	22.0	7.7
	Mauritius	AFR	6.5	48.8	13.3
	Namibia	AFR	1.7	29.5	18.7
	Peru	AMR	3.4	47.9	3.3
	Suriname	AMR	8.1	25.9	16.2
	Thailand	SEAR	6.1	25.6	6.7

AFR, African Region; AMR, Region of the Americas; EMR, Eastern Mediterranean Region; EUR, European Region; SEAR, South-East Asia Region; WPR Western Pacific Region. Data are percentage.

## Discussion

In the present large multi-country study on adolescents aged 12–15 years from LMICs, nearly half of the students were exposed to non-daily or daily secondhand smoke, with over 15% being exposed to secondhand smoke on a daily basis. Overall, while non-daily SHS was not significantly associated with obesity, adolescents who reported daily SHS had a significant 1.19 (95% CI = 1.06–1.34) times higher odds of obesity than those who reported no exposure to secondhand smoke. The strengths of this study include the large sample size and the use of nationally representative data of adolescents attending school. To the best of our knowledge, this is the first multi-country study on SHS and obesity from LMICs, and also the largest study on this topic to date.

The prevalence of obesity in our study (3.9%) was lower than the global age-standardized prevalence of obesity estimated in the Global Burden of Disease study, which reported a prevalence of 5.6% and 7.8% in 2016 among girls and boys, respectively.^16^ This may have been attributable to the fact that our study was based on data from LMICs, while it was restricted to those who do not smoke. The findings on SHS and obesity of our study are in line with previous studies from HICs that have found a positive association between SHS and greater BMI, mostly in young children. Our study results further add to the existing literature by showing that SHS is associated with obesity among young adolescents in a variety of LMICs. Although the mechanisms linking SHS and obesity are not completely understood, several mechanisms have been suggested. First, insulin resistance and metabolic imbalance can be induced by compounds found in smoke.^10^ Second, previous research has also shown that SHS is an independent risk factor for inflammation and oxidative stress,^11^ and this could indirectly favor the occurrence of obesity.^12^

Alternatively, it is also possible that the association is at least partly explained by factors that were not measured in our study. For example, maternal smoking during pregnancy could be related with both SHS and obesity in the offspring.^27^ However, a previous study showed that the association between SHS and obesity persisted even after adjustment for this factor.^9^ Next, there may be some level of residual confounding due to parental education. Children with parents with low education may be more likely to be exposed to secondhand smoke as parents with lower education may be more likely to smoke,^28^ while they also may be less likely to make an effort to avoid SHS for their children due to lack of knowledge on the health hazards of SHS. These parents may also be more likely to provide energy-dense and less healthy food to their children,^29^ and this may lead to greater body weight. Finally, the association may also be explained by parental obesity. One study showed that higher BMI is associated with higher risk for smoking among adults.^30^ Thus, if parents with obesity are more likely to smoke, this may increase the risk for SHS as well as obesity in the child as obesity is highly heritable.^31^

The study results should be interpreted in light of several limitations. First, all the variables used in our study apart from BMI were based on self-reported data. Thus, the data may be subject to biases, such as social desirability bias and recall bias. Second, the measure of SHS only referred to the number of days in which the adolescent was exposed to secondhand smoke and may not be an accurate reflection of the intensity of exposure. Furthermore, our study was based on self-report of SHS and we lacked data on objective measures (eg, cotinine). However, a previous study showed that self-reported and objective data provide consistent associations in terms of the association between SHS and obesity.^9^ Next, our data on dietary habits were limited and only consisted of fruit and vegetable consumption. Future studies should include a more comprehensive dietary assessment, including consumption of energy-dense foods. In addition, we used relatively recent data, but it is possible that our results may not reflect the current situation in LMICs, especially in countries where policies that target obesity and tobacco consumption have been developed in recent years. Finally, given the cross-sectional nature of the study, temporal associations or causality cannot be established.

In conclusion, the prevalence of SHS among nonsmoking adolescents was high, and daily SHS was associated with higher odds of obesity in LMICs. Our study results tentatively suggest that SHS prevention may have a preventive role in obesity. Future longitudinal studies may provide insight into causality and whether preventing SHS can reduce the risk of obesity. Although causality could not be established in our study, the mere fact that adolescents with obesity are more likely to be exposed to secondhand smoke is an important finding given that both SHS and obesity are major risk factors for noncommunicable diseases.^32,33^ Thus, future research should explore whether it is possible that their combined effects at youth may increase risk for early morbidity and mortality.

## Supplementary Material

A Contributorship Form detailing each author’s specific involvement with this content, as well as any supplementary data, are available online at https://academic.oup.com/ntr.

Figure S1. Country-wise association between number of days exposed to secondhand smoking in past 7 days (exposure) and body mass index (outcome) estimated by multivariable linear regression. Abbreviation: CI Confidence interval. Models are adjusted for age, sex, socioeconomic status (food insecurity), physical activity, and low fruit/vegetable intake. Overall estimate was obtained by meta-analysis with fixed effects.

ntaa053_suppl_Supplementary_Figure_S1

ntaa053_suppl_Supplementary_Figure_Legends

ntaa053_suppl_Supplementary_Taxonomy_Form

## Funding

Ai Koyanagi’s work is supported by the PI15/00862 project, integrated into the National R + D + I and funded by the ISCIII - General Branch Evaluation and Promotion of Health Research - and the European Regional Development Fund (ERDF-FEDER).

## Declaration of Interests

None declared.

## Data Sharing

The datasets supporting the conclusions of this article are available at www.cdc.gov.
