# Supplementary figures and images for: Secondhand Smoking and Obesity Among Nonsmoking Adolescents Aged 12–15 Years From 38 Low- and Middle-Income Countries
Source: Nicotine Tob Res. 2020 Mar 25;22(11):2014–21. doi: 10.1093/ntr/ntaa053 (PMC7593363; doi:10.1093/ntr/ntaa053)

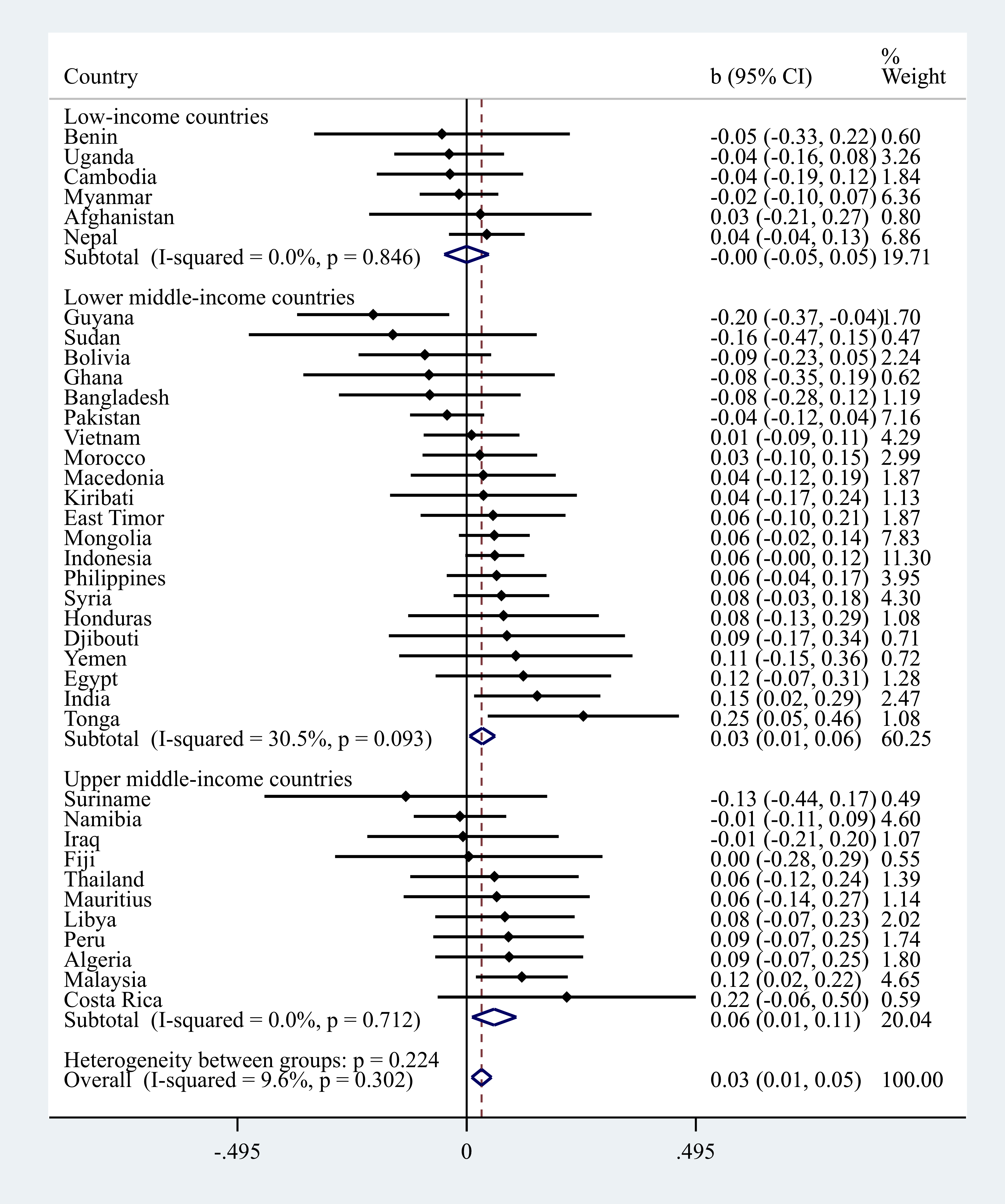

Supplement: ntaa053_suppl_Supplementary_Figure_S1 [file ntaa053_suppl_supplementary_figure_s1.jpeg]
