# Supplementary material for: Secondhand Smoking and Obesity Among Nonsmoking Adolescents Aged 12–15 Years From 38 Low- and Middle-Income Countries
Source: Nicotine Tob Res. 2020 Mar 25;22(11):2014–21. doi: 10.1093/ntr/ntaa053 (PMC7593363; doi:10.1093/ntr/ntaa053)
Supplement: ntaa053_suppl_Supplementary_Figure_Legends [file ntaa053_suppl_supplementary_figure_legends.docx]

**Figure S1** Country-wise association between number of days exposed to second-hand smoking in past 7 days (exposure) and body mass index (outcome) estimated by multivariable linear regression

Abbreviation: CI Confidence interval.

Models are adjusted for age, sex, socioeconomic status (food insecurity), physical activity, and low fruit/vegetable intake.

Overall estimate was obtained by meta-analysis with fixed effects.
